# Efficacy of Chronic Antidepressant Treatments in a New Model of Extreme Anxiety in Rats

**DOI:** 10.1155/2011/531435

**Published:** 2011-07-26

**Authors:** Hervé Javelot, Luisa Weiner, Roxane Terramorsi, Catherine Rougeot, Robert Lalonde, Michaël Messaoudi

**Affiliations:** ^1^Neuropsychopharmacology Department, ETAP-Applied Ethology, 54500 Vandoeuvre-lés-Nancy, France; ^2^Service Pharmacie, Etablissement Public de Santé Alsace Nord, 67170 Brumath, France; ^3^Laboratoire de Nutrition Génétique et Exposition aux Risques Environnementaux, INSERM U954, Service de Microscopie Electronique, Faculté de Médecine de Nancy, UHP, 54500 Vandoeuvre-les-Nancy, France; ^4^Service de Psychiatrie II, CHU de Strasbourg, 67000 Strasbourg, France; ^5^Groupe Pharmacologie Moléculaire et Intégrative, Unité de Biochimie Structurale et Cellulaire, Département de Biologie Structurale et Chimie, Institut Pasteur, 75015 Paris, France; ^6^CHUM/St-Luc, Neuroscience Research Unit, 1058 St-Denis Street, Montréal, PQ, Canada H2X 3J4

## Abstract

Animal models of anxious disorders found in humans, such as panic disorder and posttraumatic stress disorder, usually include spontaneous and conditioned fear that triggers escape and avoidance behaviors. The development of a panic disorder model with a learned component should increase knowledge of mechanisms involved in anxiety disorders. In our ethological model of extreme anxiety in the rat, forced apnea was combined with cold water vaporization in an inescapable situation. Based on the reactions of vehicle controls, behaviors involved in paroxysmic fear were passive (freezing) and active (jumping) reactions. Our results show that subchronic fluoxetine (5 mg/kg, IP, 21 days) and imipramine (10 mg/kg, IP, 14 days) administration alleviated freezing and jumping behaviors, whereas acute fluoxetine (1 mg/kg, IP) provoked opposite effects. Acute low dose of diazepam (1 mg/kg, IP) was not effective, whereas the higher dose of 3 mg/kg, IP, and clonazepam (1 mg/kg, IP) only had an effect on jumping. Paroxysmic fear generated in this experimental condition may therefore mimic the symptomatology observed in patients with anxiety disorders.

## 1. Introduction

Rodents' defensive behaviors are often studied in relation to human psychopathology, such as generalized anxiety disorder (GAD), panic disorder (PD), and posttraumatic stress disorder (PTSD). These behaviors consist of immediate defensive reactions connected with the flight or fight system and in anticipatory defensive behaviors, such as risk assessment and neophobic responses [1].

There seems to be a relationship between risk assessment and GAD, on one hand, and escape behaviors and PD on the other [2–4]. Whereas escape behaviors tend to occur in relation to a proximal threat, freezing is connected with distant threats. Both reactions allow a remotely located prey to avoid being detected and to prepare flight or fight responses when confronted with a predator [5–7]. It is noteworthy that wild rodents tend to flee, while laboratory-bred rats tend to freeze [8]. Escape is recognized as the most relevant equivalent of panic attacks in the mouse defensive test battery (MDTB) [2], the unstable elevated exposed plus-maze (UEEPM) [9, 10], and the elevated T-maze [11]. This behavior is also used in the model of dorsal periaqueductal gray stimulation (dPAG), which appears as a largely valid model for panic [12–17]. Moreover, recent studies using chemical or electrical stimulation, such as inhibition of dorsomedial hypothalamus [18, 19] and stimulation of dPAG, suggest that these structures are involved in anxiety disorders [20–25].

Subchronic administration of fluoxetine (FLX), a panicolytic agent in humans, reduced freezing caused by dPAG [26] and contextual fear conditioning [27]. Flight is a crucial response during confrontations with a predator [28] and constraining environments, for example, in a natural disaster [1]. When confronted with earthquakes, fires, or floods, panic can be predominant [29–31]. Following Caroline and Robert Blanchard's work on the visible burrow system [28] and the MDTB [32], we propose a constraining environment with a sudden rise of water level akin to flooding of a burrow and underwater trauma-induced stress [33]. Near drowning elicited a more severe response than exposure to a predator's scent [34]. This tangible life-threatening situation may model acute and chronic reactions to stress. 

Several data are consistent with our new model. For example, Bouwer and Stein showed an association between PD and a traumatizing suffocation event [35]. Severson et al. indicate that midbrain 5-hydroxytryptamine (5-HT) neurons are central pH chemoreceptors [36] and patients with PD have a hypersensitive chemoreceptor system [37] and persistent respiratory difficulties [38]. Moreover, Bouton et al. emphasize the role of conditioning in the development of PD [39].

The present study provides a new ethological model of escape and freezing attempts in rats due to suffocation fear.

The primary aim of our study was to evaluate active (escape attempts) and passive (immobility/freezing) responses to rising water levels and to determine the efficacy of antipanic or anxiolytic agents. FLX is a selective 5-HT reuptake inhibitor and imipramine (IMI) a combined noradrenaline and 5-HT reuptake inhibitor both used for treating chronic anxiety disorders, such as PD [40–43] and PTSD [44–47]. Contrary to their panicolytic effects after chronic treatment, 5-HT reuptake blockers sometimes cause a panicogenic effect after acute administration [48–50]. Diazepam (DZP) was also tested for its value in treating GAD [51, 52], but to a lesser extent PD and PTSD. Finally, clonazepam (CZP), a high-potency benzodiazepine, is frequently used in the treatment of PD because of its rapid action onset and its good tolerability [53, 54].

## 2. Materials and Methods

### 2.1. Animals

Naive male Wistar/Han rats, weighing 280–300 g at testing onset, were obtained from Harlan (The Netherlands). Prior to testing, the rats were housed in a regulated environment (humidity 50 ± 5%; temperature 22 ± 2°C; lights on 20:00–08:00). They were allowed free access to food (food pellets 2016, Teklad, USA) and tap water *ad libitum*. After an acclimatization period of 7 days, the rats were weighed and randomly distributed in treatment groups. The present protocol respects the guidelines provided by the ASAB Ethical Committee for the treatment of animals in behavioral research and teaching (Animal Behavior 2006, 71, 245–253), by the Canadian Council on Animal Care (Guide to the Care and Use of Experimental Animals: Vol. 1, 2nd Edn., 1993, vol. 2, 1984), and by the European Communities Council Directive of 24 November 1986 (86/609/EEC).

### 2.2. Drugs

FLX, IMI, DZP, and CZP were purchased from Sigma, France. All drugs were administered in a volume of 1 mL/kg body weight. Separate groups of animals were used in the evaluation of aversive behaviors. The effects of FLX (5 mg/kg, IP) and IMI (10 mg/kg, IP) were assessed after subchronic administration of 21 and 14 days, respectively. On test day, FLX and IMI were administered 30 min before testing. DZP (1 and 3 mg/kg, IP), CZP (1 mg/kg, IP), and FLX (10 mg/kg, IP) were assessed after acute administration 30 min before testing. Doses were chosen on the basis of previous results in anxiety or panic models: for DZP [26, 55], CZP [3], FLX [18, 55], and IMI [56, 57]. FLX and IMI were dissolved in a 0.9% saline solution, DZP and CZP in a 40% propylene glycol-10% ethanol vehicle. The control groups were given a 0.9% saline solution for experiments with FLX and IMI and a 40% propylene glycol-10% ethanol solution for DZP and CZP.

### 2.3. Apparatus

The apparatus consisted of a transparent Plexiglas cylinder (diameter 20 cm, height 60 cm) placed on a glass plate. Above the cylinder protruded a shower pommel connected to a tap for water delivery at 15°C. In the “Intermittent cold water swim stress” paradigm, Christianson and Drugan [58] used this temperature and their pilot studies indicated that 15°C was the lowest temperature that did not harm the rats' health. In the “Stress by immersion in cold water” Retana-Márquez et al. model [59], rats were placed in a tank of water at the same temperature.

Two types of cylinders were employed. In habituation and test sessions, the cylinder contained a hole, allowing water drainage and the possibility of jumping (height of water level 10 cm). In conditioning sessions (see below), water was accumulated as the hole was closed up.

### 2.4. Procedure

**Figure 1:**
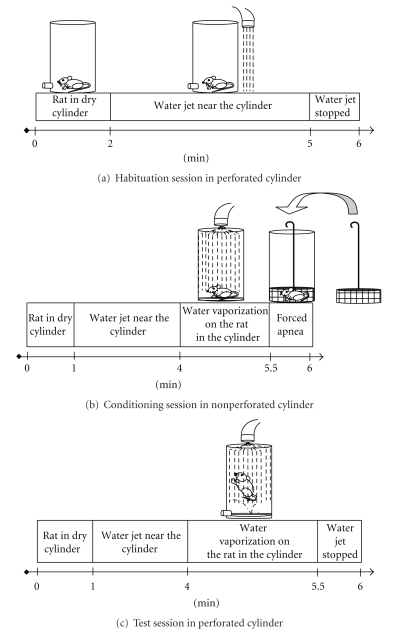
Schematic representation of the procedure used for assessment of behavioral responses. (a) Habituation session in perforated cylinder. (b) Conditioning session in nonperforated cylinder. (c) Test session in perforated cylinder.

The paradigm comprised 6-test sessions of 6 min: two habituation sessions (morning and afternoon of day 1), two conditioning sessions (morning and afternoon of day 2), and two test sessions (baseline before treatments and test after treatment administration) (Figure 1). The baseline session took place in the morning of day 3, and the test session was held either in the morning of day 4 for acute assessment or a few days later, also in the morning, for subchronic assessment. 

In the habituation session, the rat was placed inside the dry cylinder and water was delivered during a 2 to 5 min period on the glass plate beside the cylinder. The rat was left for an additional min and then returned to its home cage (Figure 1(a)). 

In the conditioning session, the rat was placed inside the dry cylinder again for 1 min. During the following 3 min, water was jet propulsed on the glass plate. From the fourth min on, water was vaporized on the rat for 90 s. Water delivery was then stopped and the rat underwent a 30 s period of partial apnea by closing the top of the cylinder with a perforated lid. The time spent underwater was based on the “underwater procedure” described in Richter-Levin's underwater trauma model [33], in which rats swim for 1 min in a water maze [60] without an escape platform and then are forcibly held under water for 30 s by a metal net. In our model, at the end of the partial apnea phase, the rat was dried off with paper towels and then returned to its home cage (Figure 1(b)). In the test session, the same procedure was repeated, except that no forced apnea was applied (Figure 1(c)). 

Rat behaviors were video recorded during test sessions and scored by experimenters unaware of treatment variables. The number and latencies of jumps were measured, together with freezing time, defined by immobility for at least 4 s in the interval of 0 to 4 min.

### 2.5. Statistical Analyses

The Mann-Whitney *U* test was used in order to compare group effects. For repeated measures, the Wilcoxon test was used. Data are expressed as the median with limits of interquartile range values, and the level of significance is fixed at *P* < 0.05. The statistical analyses were carried out with Statview 5.0 software (SAS Institute, Carey, USA, 1992–1998).

## 3. Results

### 3.1. Subchronic Fluoxetine (5 mg/kg, IP, 21 days)

As shown in Table 1, jumping prior to injections was not different between the groups (*U* = 39.50; NS) but FLX administration decreased jumping compared with vehicle (*U* = 15.50; *P* = 0.03). While jumping frequency during baseline and test sessions was stable in controls (*z* = 0.77; NS) it decreased in FLX-treated rats (*z* = 2.37; *P* = 0.02). Likewise, immobility time between the two groups did not differ at baseline (*U* = 24; NS), but was altered after drug administration (*U* = 7; *P* = 0.003). The duration of immobility in baseline and test sessions remained stable in FLX-treated rats (*z* = 0.65; NS), but increased in controls (*z* = 2.55; *P* = 0.01).

### 3.2. Subchronic Imipramine (10 mg/kg, IP, 14 days)

As seen in Table 2, the groups did not differ in terms of jumps prior to injections (*U* = 21.50; NS). After injections, IMI-treated rats displayed fewer jumps (*U* = 3; *P* = 0.006). The number of jumps between baseline and testing was unchanged in control rats (*z* = 0.85; NS), while it decreased in IMI-treated rats (*z* = 2.03; *P* = 0.04). IMI-treated rats showed lower immobility time after injections (*U* = 6; *P* = 0.02), but not at baseline (*U* = 23; NS). Relative to baseline, immobility time did not decrease significantly in IMI-treated rats (*z* = 1.69; *P* = 0.09) while it increased in controls (*z* = 2.37; *P* = 0.02).

### 3.3. Acute Diazepam (1 mg/kg, IP)

As seen in Table 3, jumping frequencies did not differ before or after injections (*U* = 34.50; NS on baseline and *U* = 25; NS on test) and remained stable in both groups (*z* = 0.42; NS for controls and *z* = 1.72; NS for DZP-treated rats). No significant difference was observed in the duration of immobility between the two groups during baseline and test session (*U* = 37.5; NS on baseline and *U* = 21; NS, on test). The duration of immobility remained stable in DZP-treated rats between the two test sessions (*z* = 1.24; NS), while it increased in control rats (*z* = 2.07; *P* = 0.04).

### 3.4. Acute Diazepam (3 mg/kg, IP)

As seen in Table 4, duration of immobility, number of jumps, and jumping latencies were not different between vehicle and DZP groups before treatment (*U* = 45, 39.5 and 46.5; NS, resp.). During the test session, DZP-treated rats showed fewer jumps (*U* = 19.5; *P* = 0.02), the latency before the first jump was higher in this group (*U* = 19.5; *P* = 0.02), and immobility was similar in both groups (*U* = 43.5; NS). This last parameter increased significantly between baseline and test session in the vehicle and DZP-treated rats (*z* = 2.70; *P* = 0.007 and *z* = 2.80; *P* = 0.005, resp.). The number of jumps remained stable in control rats (*z* = 0.05; NS) but decreased in DZP-treated ones (*z* = 2.70; *P* = 0.007). Jumping frequencies remained stable in the two groups (*z* = 1.58; NS for controls and *z* = 1.32; NS for DZP-treated rats).

### 3.5. Acute Clonazepam (1 mg/kg, IP)

As seen in Table 5, duration of immobility, number of jumps, and jumping latencies were not different between vehicle and CZP groups at baseline (*U* = 39.5, 36.5 and 31.5; NS, resp.). During test session, CZP-treated rats displayed fewer jumps (*U* = 0; *P* = 0.0003), the latency before the first jump was higher in this group (*U* = 13; *P* = 0.01), and immobility was similar in both groups (*U* = 39; NS). The immobility duration increased significantly between baseline and test session in both groups (*z* = 2.55; *P* = 0.01 for controls and *z* = 1.95; *P* = 0.05 for CZP-treated rats). The number of jumps remained stable in control rats (*z* = 0.41; NS), while it decreased in CZP-treated rats (*z* = 2.67; *P* = 0.008). Jumping frequency remained stable in controls (*z* = 0.21; NS), whereas it increased in CZP-treated rats (*z* = 2.55; *P* = 0.01).

### 3.6. Acute Fluoxetine (1 mg/kg, IP)

As seen in Table 6, the results from acute FLX rats differed from those of chronic FLX animals. Jumping frequencies did not differ before or after injections (baseline: *U* = 34.50; NS and test: *U* = 38; NS). While jumping frequency remained stable in controls (*z* = 1.72; NS), it increased in FLX-treated rats (*z* = 2.31; *P* = 0.02). The duration of immobility was not different between the two groups during baseline and test sessions (baseline: *U* = 27; NS and test: *U* = 20; NS). However, this variable increased in FLX-treated rats and controls (*z* = 2.31; *P* = 0.02 and *z* = 1.96; *P* = 0.05, resp.).

## 4. Discussion

Panic disorder is characterized not only by the presence of unexpected and recurring panic attacks, but also by a persistent and intense fear of further attacks. According to Klein [61], Barlow's psychological model of panic attacks [62] suggests that they are related to an oversensitiveness to CO_2_ [63, 64] which was later confirmed in clinical-setting studies [65]. This oversensitiveness could be explained by a disturbed warning system involved in suffocation fear. The false alarm leads to acute dyspnea, fear of impending death, and an urgent need to flee. Models of panic using pharmacological agents, such as sodium lactate, 5% CO_2_, or doxapram (respiratory analeptic), induce false suffocation alarms, similar to those found in panic attack in terms of physiological specificity and pharmacological reactivity [38, 61, 66–70], and doxapram has been also used in the rodent to determine its neuroanatomic basis [71].

The behaviors observed during panic attacks (flight, acute dyspnea) and the experimental conditions that we have developed in the forced apnea test define the face validity of our model. The combination of 30 s forced apnea and cold water appears necessary in our model to induce a chronic stress reaction with significant behavioral expression related to paroxystic fear (unpublished data).

### 4.1. Task Parameters

Two stressors were used cold water and restraint/immobilisation—in order to model extreme fear conditions. Cold stress is a well-documented stressor in rats [72–74]. For example, Jedema and Grace [75] demonstrated activation of locus coeruleus neurons after exposure to low temperatures in rats, thus central noradrenergic function seems potentially modifiable in anxiety. Hyperventilation and tachycardia occur before cold water immersion in humans as a form of anticipatory anxiety [76], and the “cold shock” itself causes an “inspiratory gasp,” hyperventilation and secondary dyspnea, hypocapnia, tachycardia, and hypertension [77]. These somatic symptoms are akin to spontaneous manifestations during panic attacks [61].

The main purpose of our study was to create ethological fear sequences through flooding inside a rodent's burrow. Combined restraint and cold stressors are relevant in inducing chronic stress conditions in rats [78, 79]. Restraint/immobilisation combined with cold water immersion produces more behavioral alterations than immobilisation alone [80]. Likewise, Retana-Márquez et al. [59] showed stressful effects induced by immersion in cold water, both acutely and chronically. 

The number of jumps, the latency before the first jump, and the immobility time were assessed in male adult rats exposed to our model. In placebo controls, paroxysmic fear induced both active (jumps) and passive (immobility) reactions. At baseline, all rats jumped every 3.5 s during vaporisation, indicating that this is a typical reaction to a proximal threat. The same animals spent 87.5% of their time in a freezing posture during application of water beside the cylinder, suggesting that this models a typical reaction to a distant threat. Richter-Levin [33] used water trauma in a model of PTSD. Similarly, classical conditioning is probably involved in PD [39]; Bouwer and Stein [35] showed a relationship between PD and near drowning. Taken together, these data support the idea that severe anxiety disorders and panic attacks may be mimicked in laboratory settings and still be ethically acceptable because of their brief duration.

### 4.2. Subchronic Fluoxetine and Imipramine

Subchronic administration of FLX and IMI caused similar effects. Both substances decreased jumps and immobility time and increased time latency before the first jump. All these three effects are attributable to their anxiopanicolytic properties. They concur with those of PD paradigms. With the MDTB, Griebel et al. [56] reported a significant decrease in the number of mouse escapes from a predator (anaesthetised rat) after FLX treatment for 21 days at the dose of 5 mg/kg IP. Likewise, in the UEEPM [9], the number of escapes of FLX-treated rats (10 mg/kg, PO) was lower than that of controls. Moreover, Vargas and Schenberg [81] showed that 3-week FLX treatment at the dose of 5 mg/kg, IP, increased dPAG stimulation thresholds causing escape attempts. Similarly, various selective serotonin reuptake inhibitor (SSRIs), such as citalopram, FLX, paroxetine, sertraline, and escitalopram, reduced the flight-like escape behavior produced by dPAG electrical stimulation in the rat [12, 13]. Borelli et al. [26] found that 2-week FLX treatment at 5 mg/kg, IP, increased dPAG stimulation thresholds for freezing, but not escape attempts. The latter data, as our own, indicate that freezing is a relevant measure in animal models of extreme anxiety, although the main characteristics of panic is the flight response. These results suggest that FLX decreases freezing responses in rats under intense fear conditions. They are also consistent with the results obtained by Santos et al. [27]. Moreover, the CCK-4 (cholecystokinin type 2 (CCK(2)) receptor agonist) intradorsolateral periaqueductal gray injection facilitated the expression of both freezing and escape behaviors [14]. These data support the hypothesis that both locomotor reactions are closely related to panic behaviors and should be taken into account given their high expression levels in our model.

At the pharmacological level, chronic administration of SSRIs treatments appears to sensitize 5-HT1A receptors in the dPAG and supports the idea that facilitation of 5-HT1A receptor-mediated neurotransmission in the dPAG is implicated in the pharmacotherapy of PD [13, 16, 17].

Finally, FLX, paroxetine, and sertraline SSRIs are also effective in different models of PTSD in rats [82–84]. 

Tricyclic agents, such as IMI at 10 mg/kg/14 d, had similar effects to those obtained with FLX at 5 mg/kg/21 d in terms of escape attempts and freezing duration. In the MDTB “predator avoidance test,” IMI at 5 and 10 mg/kg for 21 days decreased mouse avoidance distance and frequency of escapes from the rat [56]. Likewise, Jacob et al. [85] showed that a 3-week IMI treatment at 15 mg/kg, IP, produced an enhancement of the antiaversive effect of 5-HT receptor agonists locally injected into the PAG. Blanchard et al. [86] observed a decrease of freezing in rats after presentation of a cat after 3-week IMI treatment at 15 mg/kg, IP. Our results are reminiscent of successful PD and PTSD treatments [40–47] after chronic administration of IMI or FLX in clinical studies.

### 4.3. Acute Benzodiazepines

DZP is not considered to be as effective as other antipanic agents [87]. At 1 mg/kg, DZP only prevents the increase of immobility after fear conditioning and has no effect on escape attempts. Likewise, no effect was observed after PAG stimulation with DZP at the doses of 1, 2, and 4 mg/kg, IP [26] and one-way escape was not affected by DZP at 0.5, 1, 2, and 4 mg/kg, IP in the elevated T-maze [11]. However, Griebel et al. [55] found a panicolytic effect with DZP at 3 mg/kg in the MDTB but not at 0.5 and 1 mg/kg, IP. In our model, DZP, at 3 mg/kg, IP, decreases the number of jumps, without affecting the duration of immobility as it was previously noted with a lower dose. Our interpretation of these results is that, at 1 mg/kg, IP, DZP only acts on anticipatory anxiety (freezing), without interacting with panic reaction (jumps), whereas, at 3 mg/kg, IP, its sedative effects mask its potential effect on anxiety, but is able to decrease panic-related symptoms. Li et al. [88] evaluated the effects of DZP in a PTSD model—consisting of a 2-day foot shock (0.8 mA, 10 s) period followed by 3 weekly situational reminders. After 26-day IP administration, DZP at a low dose of 0.25 mg/kg, but not at 4 mg/kg, reduced behavioral deficiencies. Additional data is therefore needed concerning possible benzodiazepine effects at variable doses, such as subchronic administration and a wider range of doses used in our paradigm. Although benzodiazepines are used in the short-term treatment of PTSD, long-term treatment with these molecules is not effective [89–91].

In addition, a 3-day treatment with CZP at 5 mg/kg, IP, completely blocked the effects of bicuculline following its infusion in dorsomedial hypothalamus [19]. After acute CZP administration at 0.1, 0.56, and 1 mg/kg, IP, Jenck [92] noted that it reduced, in a dose-related manner, aversive behaviors induced by dPAG stimulation. In the MDTB, CZP single doses of 0.3 and 1 mg/kg, IP, decreased avoidance distance and avoidance reactions [86]. Our results are consistent with those of Blanchard regarding DZP admnistration at 3 mg/kg, IP, but not following FLX and IMI administration. In our study, CZP only affected the number of jumps. Unlike our results following DZP admnistration, a significant increase of latency before the first jump was observed between baseline and test after CZP intake. This effect can be explained by CZP's superior efficacy on panic attack symptoms compared to DZP. Conversely, CZP did not prevent the increase of immobility after forced apnea. These results are consistent with the clinical data suggesting that CZP reduces panic attack intensity but has no effect on anticipatory anxiety in PD [93].

At the pharmacological level, alprazolam facilitates 5-HT1A receptor-mediated neurotransmission in the dPAG, like SSRIs [15]. This could partially explain the similar effects obtained by SSRIs, high-potency benzodiazepines, such as alprazolam and clonazepam, and low-potency benzodiazepine, such as diazepam at sedating doses, on flight-like escape behaviors.

### 4.4. Acute Fluoxetine

The panicogenic effect of acute FLX is a well-known parameter in clinical practice [49] or in animal experimentation [3]. In the MDTB, acute FLX increased avoidance distance [56]. However, in the UEEPM, acute FLX did not increase rodent escape behavior [9], and in dPAG stimulation models, acute FLX either attenuated escape behaviors [94] or had no effect on these behaviours [26]. In our model, FLX at 10 mg/kg, IP, had a panicogenic effect which was measured via the comparison of two ethological variables between baseline and test session in FLX-treated rats, that is, increase of the number of jumps and decrease of latency before the first jump. 

In conclusion, our model is an innovative behavioral paradigm that may improve investigation of anxiety disorder. Our results are consistent with the hypothesis that effective drugs for GAD and PD/PTSD have differential effects on specific defensive behaviors in rats. Antidepressant agents, such as IMI and FLX, counteract anticipatory anxiety and panic symptoms, whereas high potency benzodiazepines or low potency benzodiazepines at sedating doses only affect the panic-related symptoms. 

Clinical observations also seem to converge with our results. Indeed, benzodiazepines immediately decrease panic attack-related anxiety symptoms; however, antidepressant drugs remain the gold standard treatment for the long-term management of PD [95].

## Figures and Tables

**Table 1:** Effects of subchronic fluoxetine (FLX) treatment (5 mg/kg/21 d, IP, *n* = 9, median with limits of interquartile range values). Mann-Whitney *U* test: *P < 0.05; **P < 0.01  (FLX versus Vehicle). Wilcoxon-test: ^#^ *P* < 0.05 (test versus baseline). Data were expressed as median with limits of interquartile range values.

Measures	Baseline (before treatment)		Test (after treatment)	
	Vehicle (*n* = 9)	FLX (*n* = 9)	Vehicle (*n* = 9)	FLX (*n* = 9)
Number of jumps	23 (9.8–37.3)	21 (16.5–31.5)	22 (10.5–27)	9^∗#^ (0–12.8)
Latency before the first jump (s)	15 (11.3–21.5)	14 (11.8–23.3)	16 (12.3–18)	27^∗#^ (16.3–90)
Immobility (s)	140 (126.8–144.8)	59 (33.3–93.8)	164^#^ (140–176.8)	70** (58.8–115)

**Table 2:** Effects of subchronic imipramine (IMI) treatment (10 mg/kg/14 d, IP, *n* = 7, median with limits of interquartile range values). Mann-Whitney *U* test: *P < 0.05; **P < 0.01 (IMI versus vehicle). Wilcoxon-test: ^#^ *P* < 0.05 (test versus baseline). Data were expressed as median with limits of interquartile range values.

Measures	Baseline (before treatment)		Test (after treatment)	
	Vehicle (*n* = 7)	IMI (*n* = 7)	Vehicle (*n* = 7)	IMI (*n* = 7)
Number of jumps	24 (17.3–29)	22 (15.8–25)	28 (24.5–32.5)	15^∗∗#^ (1.8–18.8)
Latency before the first jump (s)	5 (3.3–11)	12 (6.3–24.3)	10 (7.3–18)	22^∗∗#^ (19.8–74.5)
Immobility (s)	107 (93.5–117)	103 (93.5–150.8)	157^#^ (124.8–182.5)	86* (33.5–128.5)

**Table 3:** Effects of acute diazepam (DZP) treatment (1 mg/kg, IP, *n* = 9, median with limits of interquartile range values). Wilcoxon-test: ^#^ *P* < 0.05 (test versus baseline). Data were expressed as median with limits of interquartile range values.

Measures	Baseline (before treatment)		Test (after treatment)	
	Vehicle (*n* = 9)	DZP (*n* = 9)	Vehicle (*n* = 9)	DZP (*n* = 9)
Number of jumps	20 (15.5–23.5)	21 (15.3–28.8)	21 (17.5–23.3)	16 (12.3–22.3)
Latency before the first jump (s)	17 (9.8–21.8)	15 (10.8–20.8)	13 (9.8–15.5)	21 (14.0–23.3)
Immobility (s)	106 (61.5–125.5)	101 (80.3–129.8)	121^#^ (104.0–141.3)	90 (75.0–103.0)

**Table 4:** Effects of acute diazepam (DZP) treatment (3 mg/kg, IP, *n* = 9, median with limits of interquartile range values). Mann-Whitney *U* test: *P < 0.05 (DZP versus vehicle). Wilcoxon-test: ^##^ *P* < 0.01 (test versus baseline). Data were expressed as median with limits of interquartile range values.

Measures	Baseline (before treatment)		Test (after treatment)	
	Vehicle (*n* = 9)	DZP (*n* = 9)	Vehicle (*n* = 9)	DZP (*n* = 9)
Number of jumps	13.5 (12–17)	15.5 (8–28)	15 (10–19)	6^∗##^ (4–14)
Latency before the first jump (s)	15.5 (12–25)	23.5 (6–34)	11 (6–20)	28* (12–78)
Immobility (s)	84 (44–129)	85.5 (52–120)	129.5^##^ (90–176)	130^##^ (83–171)

**Table 5:** Effects of acute clonazepam (CZP) treatment (1 mg/kg, IP, *n* = 9, median with limits of interquartile range values). Mann-Whitney *U* test: *P < 0.05; **P < 0.01 (CZP versus vehicle). Wilcoxon-test: ^#^ *P* ≤ 0.05; ^##^ *P* < 0.01 (test versus baseline). Data were expressed as median with limits of interquartile range values.

Measures	Baseline (before treatment)		Test (after treatment)	
	Vehicle (*n* = 9)	CZP (*n* = 9)	Vehicle (*n* = 9)	CZP (*n* = 9)
Number of jumps	17 (9.75–21.75)	15 (11–17)	18 (15.25–20.5)	3^∗∗##^ (2–8)
Latency before the first jump (s)	14 (6.5–35.75)	12 (4.5–19.5)	10 (7.25–31)	68^∗#^ (20.5–75)
Immobility (s)	90 (50–133.5)	85 (65.25–115)	130^#^ (92–162)	125^#^ (104–145.5)

**Table 6:** Effects of acute fluoxetine (FLX) treatment (10 mg/kg, IP, *n* = 9, median with limits of interquartile range values). Wilcoxon-test: ^#^ *P* < 0.05 (test versus baseline). Data are expressed as median with limits of interquartile range values.

Measures	Baseline (before treatment)		Test (after treatment)	
	Vehicle (*n* = 9)	FLX (*n* = 9)	Vehicle (*n* = 9)	FLX (*n* = 9)
Number of jumps	17 (13.5–19.5)	15 (13.3–17.8)	19 (16.8–24.8)	21^#^ (19.0–22.5)
Latency before the first jump (s)	19 (12.8–27.3)	20 (14.8–29.5)	16 (13.0–17.8)	14^#^ (4.8–19.5)
Immobility (s)	85 (68.0–98.0)	97 (81.5–114.8)	108^#^ (93.8–126.3)	139^#^ (115.0–159.3)
